# “Once the child is delivered, he is no more your baby,” Exclusive Breastfeeding experiences of first-time mothers in Kassena-Nankana Municipality, Ghana - a qualitative study

**DOI:** 10.1186/s12884-020-03272-5

**Published:** 2020-09-29

**Authors:** Louisa Adda, Kwabena Opoku-Mensah, Phyllis Dako-Gyeke

**Affiliations:** Department of Social and Behavioral Sciences, School of Public Health, College of Health Sciences, University of Ghana, P.O. Box LG 13, Legon, Ghana

**Keywords:** First-time mothers, Exclusive breastfeeding, Perceptions

## Abstract

**Background:**

Exclusive Breastfeeding (EBF), for the first 6 months of life, is globally accepted as the preferred method for infant feeding. In Ghana, an estimated 84% of children < 2 months old are exclusively breastfed.

But by age 4 to 5 months, only 49% continue to receive EBF. This situation continues to deteriorate. Thus, the need to explore perceptions, practices as well as factors that influence EBF in Ghana.

**Methods:**

Using a qualitative design, four focus group discussions were conducted among first-time mothers and eight in-depth interviews with health workers and traditional birth attendants. The study was conducted in four communities in the Kassena-Nankana municipality of Ghana. Discussions and interviews were recorded and later transcribed verbatim to English language. The transcribed data was then coded with the aid of analysis computer software (Nvivo version 10.0) and later analyzed for the generation of themes.

**Results:**

Exclusive breastfeeding is practiced among first-time mothers due to its perceived benefits; which include nutritional advantage, ability to enhance growth whilst boosting immunity and its economic value. However misconceptions as well as, certain cultural practices (e.g. giving herbal concoctions, breastmilk purification rites), and relational influences, may threaten a mother’s intention to exclusively breastfeed. Relational influences are mainly from mother in-laws, traditional birth attendants, grandmothers, herbalists and other older adults in the community.

**Conclusions:**

Although first time mothers attempt EBF, external influences make it practically challenging. The availability and utilization of information on EBF was found to positively influence perceptions towards EBF, leading to change in attitude towards the act. Thus, the practice of community-based health services may be strengthened to provide support for first-time mothers as well as continuous education to the mother in laws, female elders and community leaders who influence decision making on breastfeeding of infants.

## Background

Globally, about 90% of infants in low and middle-income countries (LMICs) located in Africa, South Asia, and Latin America were breastfed for at least 12 months [1]. However, only 21.3million infants (37%) aged less than six months are exclusively breastfed in LMICs [1]. Although the practice has been globally acknowledged as the preferred method of feeding infants, West African countries continue to record lower rates of exclusive breastfeeding (EBF) [1–3]. According to World Health Organization (WHO), EBF entails giving only breast milk from a mother or wet nurse or expressed to a child, without any additional food or liquid for six months, with the exception of oral rehydration solution, or syrups of vitamins, minerals or medicines [2]. EBF has further been found to be adequate in both quality and quantity in terms of energy, protein, water and other nutrients required for the development of the infant whether the mother is healthy or undernourished [4, 5].

Consequently, WHO and United Nations Children’s Fund (UNICEF) recommend that all mothers should breastfeed their children exclusively for the first 6 months and thereafter continue to breastfeed for as long as the mother and child desire, with appropriate and sufficient weaning food included after 6 months of life [2, 3]. EBF is associated with multiple benefits ranging from cognitive to physical development over the life course of the infant [6–8]. This practice is essential for good child health in the short term given its associated lower incidence and severity of diarrhea, reduced respiratory tract infections and lower incidence of allergic diseases among children at-risk [9, 10]. In the long term, EBF has been found to predict lower incidence of obesity and cognitive impairments during childhood and adolescence [8, 11]. Studies have further shown the effect of EBF on reduced risk of other chronic diseases including; hypertension, diabetes, hyperlipidemia, hypercholesterolemia and some types of cancer in adulthood [8, 11, 12]. Similarly, women who exclusively feed are less likely to be at risk of breast cancer, diabetes, ovarian cancer and have better birth spacing [1]. It is estimated that EBF practices can prevent the projected 823,000 child mortality and 20,000 maternal mortality which results from breast cancer annually [13].

In Ghana, while breastfeeding initiation rates are encouraging, their rates are below acceptable recommendations by WHO and other international bodies [2, 3, 14]. Over the past few years, EBF has declined from 54% in 2006 to 52% in 2014, despite global and local efforts to promote the practice [15]. It was estimated in the Ghana Demographic and Health Survey Report (GDHS) that 53.1% of children aged 2–3 months are being exclusively breastfed. Unfortunately, by age 4 to 5 months, only 36.2% continue to receive EBF. The early cessation of breastfeeding to eventually use milk substitutes (e.g. water, juice, supplementation) is far too common [16]. There is also poorly timed introduction of solid, semi-solid and soft foods, often of poor quality in low resourced settings [17]. Most first-time mothers usually fall victim to these practices. Young mothers who may be inexperienced struggle with breastfeeding after delivery. First-time mothers especially are at a greater disadvantage to practice EBF when there is inadequate support. As reported in a study conducted in Vientiane, the capital city of Laos, by Lee and his colleagues, less than half of first-time mothers breastfeed for 6 months as a result of their jobs and perceptions on inadequacy of breastmilk supply [18]. Again, the unease with exposing the body in front of others as well as other psychological aspects of breastfeeding are well known [19–21].

In the context of the post-2015 development agenda, EBF is essential to achieving nine of the Sustainable Development Goals (SDGs) as well as many other international health targets [22, 23]. Child and maternal malnutrition remain a challenge in Kassena-Nankana district, in Ghana, with reported prevalence of stunting (15.6%), underweight (15.3%), maternal malnutrition (10.3%) and overweight mothers (12.4%) [24]. The potential risk for increased morbidity and under-nutrition among children, associated with early introduction of complementary foods has also been identified within the district [24]. More recently, anecdotal evidence indicates that the practices of EBF remain low especially among first-time mothers within the district. However, little is known in literature about actual EBF practices of first-time mothers and its influencing factors within this district. Thus, the aim of this study was to explore how knowledge and perceptions of EBF as well as prevailing social and cultural norms that influence the practice of EBF among first-time mothers in the Kassena-Nankana Municipality.

## Methods

### Study design, setting and participants

The study was a cross-sectional qualitative research [25]. We explored the knowledge and perceptions of first-time mothers (the primary participants) and how socio-cultural structures at the household, community and health service sector, influenced their practice of EBF. The study was conducted in the Kassena-Nankana Municipality in Northern Ghana, which has a population of about 153,000 [26]. The Municipality is located within the Guinea savannah woodlands and covers a total land of about 1675 sq. km and stretches about 55 km north-south and 53 km east-west. It is made up of 28 towns with Navrongo as its administrative capital. It has wet and dry seasons as the two climatic conditions with agriculture as the major economic activity. The Municipality has thirty-three health facilities including a government hospital, a private clinic, a health post, 25 Community Health-based Planning Services compounds (CHPS), a health research centre and 2 nutrition centres catering for the large population. Malnutrition is undoubtedly known as a major health condition in the municipality due to challenges of migration and falling standards of living [24].

Four communities in Kassena-Nankana Municipality (i.e. Pungu, Kajelo, Doba and Gaani) were purposively sampled using maximum variation sampling and snowball sampling approaches. These techniques allowed participants with diverse background (e.g. women with different socio-economic backgrounds, etc.) to be included [25, 27]. Snowball sampling was also useful because of the difficulty in obtaining the required number of eligible participants for data collection. Snowball sampling technique is another non-probability sampling technique in which the researcher begins by identifying an individual perceived to be eligible who in turn identifies other potential participants [27, 28].

First-time mothers, traditional birth attendants (TBAs) and heads of health facilities were the target population for the study. Participation was limited to primiparous women who were breastfeeding children aged 7 to 12 months. This inclusion criteria was used to target mothers who were supposed to have just completed the optimal six-month EBF [29]. This was also to help prevent the challenge of recall bias likely to be experienced among mothers who completed EBF for a longer period before the time of data collection [19].

### Data collection techniques and tools

The research team developed focus group discussion (FGD) and in-depth interview (IDI) guides (supplementary files 1 and 2). The data collection tools covered the specific objectives of the study, thus exploring knowledge, perception and practices of EBF, facilitators and barriers, as well as the coping strategies adopted by these mothers. The drafted tools were then pilot-tested after which more probe questions were added which elicited detailed responses from the participants. In-depth interviews and focus group discussions were conducted within four communities. A total of 8 IDIs and 4 FGDs were enough in reaching data saturation. Hennink and colleagues argue that four [4] focus group discussions are sufficient to reaching code saturation [30]. For the IDIs, 4 TBA interviews and 4 health worker interviews were conducted using the developed IDI guide. In each of the 4 communities, one TBA interview and one health worker interview was conducted. Furthermore, 4 FGDs were organized in the 4 communities. In 3 of the focus group discussions, participation ranged between a maximum of 12 and a minimum of 8 people, which was essential for an informative and manageable group discussion [27, 28]. However, only 5 participants in Kajelo community were recruited into the study due to the limited availability of eligible first-time mothers. Each focus group was homogenous by age, where teenage mothers and mothers aged 20 years and above were put into different groups. A key community gatekeeper was contacted to help identify the first eligible participant who in turn assisted in contacting other eligible participants.

The IDIs and FGDs were conducted at convenient times and venues with all study participants. Written informed consent was sought from all participants before the audio-recorded interviews and discussions were conducted. The principal investigator together with trained research assistants conducted all the interviews. The help of an additional assistant was obtained in conducting the interviews in Gaani community and Doba community, who translated the data collection tools into the two local languages. Each interview and FGD lasted for an average of 40 min. Field notes covering initial participant’s reactions to the interviews and FGDs, as well as relevant observations such as the demeanor of the respondent were recorded promptly after interviews and FGDs. Data gathered were stored with limited access to the research team.

Data was collected within a week in May, 2015. Measures for ensuring qualitative trustworthiness according to Lincoln and Guba were applied in this study. This included audit trail, thick description, reflexivity and member checking used to ensure credibility, dependability and transferability of the study findings [27, 28].

### Data analysis

The audiotaped interviews were transcribed verbatim. The transcripts were read over and over again and edited to remove grammatical errors before they were imported into NVivo 10.0 software for coding. A codebook was created based on the objectives of the study and the subject areas explored during the interviews. Each transcript was opened in the NVivo software and line-by-line reading and coding into nodes of all the statements were done. The coding was reviewed, where some nodes were rearranged and others merged to develop themes. As coding continued, codebook developed initially was revised. Thematic analysis was employed using both deductive and inductive approaches [15]. Major and sub-themes were identified and exemplar quotations selected for each theme. These are presented in the results section of the work.

### Ethics

Ethics approval was received from the Ghana Health Service Ethics Review Committee, Research and Development Division in Accra. Written Informed consent was obtained from all participants.

## Results

### Characteristics of participants

In all, 37 participants were involved in this study. This consisted of 29 first-time mothers who participated in the FGDs as well as TBAs [4] and health workers [4] who were a part of the in-depth interviews. Majority of the participants for the FGDs were married [28], had children aged between 7 and 9 months [19] and professed the Christian faith [27].

The majority of participants were first-time mothers with junior high school as their highest level of education [14] and they resorted to farming as their major economic activity [15]. In addition, only a few of the respondents [2] had ethnic backgrounds other than Kassena and Nankana (native groups within the study area). Table 1 provides details of the socio-demographic characteristics of the primary participants (first-time mothers). In the case of the socio-demographic characteristics of health workers interviewed, all were females, consisting of three community health nurses and one midwife who was the only one married among the four. Their years of working experience ranged between two and 6 years. Likewise, all the TBA’s interviewed [4] were females, married and occupied with farming as their major source of income. Two of the TBAs aged 42 and 58 years had middle school and primary level of education respectively whereas the other two had no formal education*.*

**Table 1:** Socio-Demographic Characteristics of First-time Mothers

Characteristics of FGD Participants	Number of participants
Ethnic Background	
Kassena	13
Nankana	14
Others	2
Age of mother	
20 years and below	14
21-26 years	15
Religion	
Christian	27
Traditionalist	2
Level of education	
No formal education	2
Primary	7
JHS	14
SHS	6
Occupation	
Artisan	3
	8
Farmer	15
Trader	4
Unemployed	7
Age of child	
7–9 months	19
10–13 months	10
Marital status	
Single	1
Married	28

Details of the study findings are structured under the following sub-headings; knowledge and perception of EBF practices, cultural perspectives on breastfeeding practices, socio-economic influencing factors for breastfeeding practices as well as changing perceptions on breastfeeding practices.

### Knowledge on EBF

We explored knowledge on EBF and its practices from the perspectives of health workers, TBAs and first-time mothers. The major source of knowledge on EBF among the mothers was health facilities where information was usually obtained after delivery. For instance, a participant made mention that, *“It was when I delivered, they [health care providers] told me about EBF practice in Navrongo, the big hospital.” (****Mother, FGD, Community 1)***. On few occasions, family members who had practiced EBF were also in the position to introduce first-time mothers to the practice as indicated by another mother, who stated that: *“I heard it [EBF] from my house women who had delivered before I got pregnant and delivered. (****Mother, FGD, Community 2)****.* Furthermore, TBAs recommended infant feeding practice, since they were another source of information on EBF to mothers who came to them for care.

Health workers, TBAs and the first time mothers also had knowledge of the benefits in practicing EBF. They perceived the practice as the initial immunity for babies against childhood diseases and infections. For instance according to one of the mothers, *“If you give your baby only breast milk for six months, he does not fall sick frequently and he will be strong”* ***(Mother, FGD, Community 4).*** Interviews with the health care providers highlighted some developmental benefits of EBF. One participant stated that *“… to the child too it [EBF] helps in especially the brain development of the child and it also helps to prevent diseases especially diarrhea in children” (****IDI, Community 1).***

EBF was also found to have a substantial advantage to women, especially because it is an effective and less costly method for family planning for nursing mothers, as observed below:

*“…it serves as a natural family planning for the woman if she practices it very well... Also if the woman doesn’t practice EBF, she’ll need to use back up methods like condoms during sex because if she doesn’t, she might get pregnant since she doesn’t breastfeed exclusively” (****IDI, Community 3).***

### Perceptions and misconceptions about breastfeeding practices

Despite the fact that first time-mothers saw the practice of EBF as vital to a child’s health and an effective practice for birth spacing and personal wellbeing of mothers, some few respondents misinterpreted the practice. To such mothers, EBF was perceived to have several disadvantages to both mother and baby, and we found such information to contrast with some earlier responses on knowledge of EBF. Several personal and cultural reasons accounted for the early introduction of babies to substitute foods and liquids. Women held the assumption on the irreplaceable nature of water required to wet the throat of babies and subsequently ease the pains associated with breast sucking. One woman expressed her worries pertaining to the comfortability of EBF saying that,

“*to be only breastfeeding the baby for six months with breast milk brings a lot of disadvantages to the mother. For instance, because you do not give the baby water and food to wet the throat, it is always dry and he sucks the breasts vigorously, which creates pains for the mother. But if you give the water, it will soften the throat and he will suck the breasts slowly” (****Mother, FGD, Community 2).***

In the same regard, some mothers believed a baby could die from thirst, if exclusively breastfed. Thus, the need to quench the thirst of babies with water was seen as a mechanism against such threat to the baby’s life. The narratives below suggest that it was not only breast milk that enhanced the health and development of babies.

*“In my opinion, it is not only breast milk that makes children grow well. We did that giving them water, they get well and become adults (****Mother, FGD, Community 2).***

In understanding how the participants perceived EBF in terms of duration and content of the practice, we found consistency in the description of the EBF information they shared. In their explanation, the first-time mothers emphasized the duration for the practice. One woman, for example, mentioned, *“Exclusive breastfeeding means, when a woman delivers, she should breastfeed the baby for six months….” (****Mother, FGD, Community 2).*** This response was similar to that of the other mothers indicating that almost all the respondents knew the duration for EBF as determined by international standards. In addition to understanding the content of EBF practice from the perspective of first-time mothers, another mother added, *“She [the mother] should not give the baby food nor water until he attains six months”* ***(Mother, FGD, Community 2)***.

We further explored participants’ perceptions on the frequency of EBF. However, our findings show a few discrepancies pertaining to the acceptable number of times a child needed to be breastfed. Some of the first time mothers were of the view that their babies demanded breast milk several times within a day while to other mothers, breastfeeding their infants for not less than 10 times in a day was seen as ideal.

### Cultural practices which inform decisions to breastfeed

The findings revealed interesting cultural complexities regarding decision making on breastfeeding practices. To a large extent, decisions on composition of infant feeds and onset and duration of breastfeeding were all culturally predetermined by existing norms within Kassena-Nankana. Although the mothers involved in the study admitted to have been taken through some form of EBF counselling and education, not all the mothers were able to strictly adhere. This was due to the existence of certain cultural structures that hindered the practice of EBF. Focus group discussions in community one for instance, revealed the culturally tainted perception on colostrum, which restricted a mother’s ability to breastfeed right after delivery. One mother mentioned the following:

*“When a woman delivered they would not allow the baby to breastfed, saying that the first breast milk was dirty. They would rather look around for a woman who had delivered some days before to come and breastfeed the newborn baby.” (****Mother, FGD, community 1).***

Also, certain rituals are often performed with the intention to test the quality of the breast milk and to purify it. It is only after these rituals are performed that a decision to breastfeed the baby was taken. This practice performed for first-time mothers and their babies is referred to as “*Kacheeri*” in *Kasem* and “*pog-saare*” in Nankani. The FGD in community two revealed that the practice involved expressing the breast milk of a mother who had just delivered into a calabash often done by the elderly women in the house, which usually includes the mother-in-law. This is to determine whether breast milk is good for consumption by the baby or not. Live ants are then put into the calabash of breast milk if the ants survive and are able to crawl out then the milk is graded as good for the baby. Otherwise, help from the herbalist is sought to purify the breastmilk and the baby is fed on other foods such as cow milk, millet flour, water and other herbal concoctions while awaiting the mother’s breast milk to be purified. Duration for the purification process was also dependent on the promptness of the herbalist’s judgement. Thus, the herbalist is a key figure in the determination of breastfeeding practices in Kassena-Nankana. During the purification period, the mother’s breast milk will always be expressed and discarded.

Furthermore, the study revealed other cultural practices intended to keep the baby strong and healthy. More specifically, herbal concoctions were given to babies to prevent certain deformities that can affect the baby’s head *(fontanels)* or hinder their ability to walk. Such practices according to first-time mothers in community two, started immediately one delivers and was discharged from the hospital to the house, as older women were quick to commence feeding the baby their herbal concoctions. Concoctions may be given for 4 or 3 days to the baby girl or boy, respectively. Some of these beliefs were known by participants:

*“I think it’s their belief; especially they are concerned with their fontanels, that if you don’t give medicine to the child, his/her head would divide” (****IDI, Community 4).***

*“They [mothers who just delivered] are however allowed to breastfeed the baby on the first day but they [mother in-laws and other elderly women] still introduce the concoctions/herbs.”*

***(IDI, Community 2).***

### Relational influences on EBF decision-making

Furthermore, the desire of a mother to exclusively breastfeed her baby could only materialize when significant family members, especially mothers-in-law consented to that decision. Thus, mother-in-laws also exerted much influence on the newborn baby and the mother. The findings indicate that mothers-in-law were the major enforcers of certain cultural formalities performed for first-time mothers and their babies upon reaching home after delivery. The study participants invariably described some mechanisms through which mothers-in-law and elderly women compelled first-time mothers to feed their babies with herbal concoctions believed for spiritual and sedative advantages. To support this assertion, one of the primary respondents highlighted that:

*“The old ladies [referring to mothers-in-law and other older women] always say that if you don’t allow them to give the baby the herbal concoctions and water to drink and the baby is crying in the night, they won’t come to your aid. They will leave you alone to take care of your baby and that, it is the herbal concoctions and water that put the baby to sleep without worrying the mother.” (****Mother, FGD, Community 3).***

Similarly, as unique to collective cultures, first-time mothers in Kassena Nankana did not have much control over their babies. Due to their inexperience and dependence the older women in taking care of their babies, they often become vulnerable and are at the mercy of the community gatekeepers for prevailing cultural norms. In addition, women were culturally expected not to act contrary to the dictates of in-laws, especially mother-in-law and other elderly women in the household. Therefore elderly women and mother in laws capitalized on this norm and restrictions on women, serving as a formalized structure to resist the practice of EBF. As was stated by one first-time mother:

*“The old ladies don’t agree at all because they bath the baby and do everything so when you want to complain [against the use of herbal concoctions], they will tell you that once the child is delivered, he is no more your baby so you cannot control them. What they want was what they will do”* ***(Mother, FGD, Community 2).***

The contribution of the key informants also confirmed how family influence played an important role in the decision-making process for EBF. Therefore, a mother’s decision to exclusively breastfeed her baby for 6 months depends on the approval of key family members. A participant, for instance, expressed the difficulty with obtaining approval to exclusively breastfeed that,

*“It’s not easy at all. For most of the mothers, their families and partners always complain because they think that the child is not only supposed to take breast milk” (****IDI, Community 3)****.*

In the same regard, *a participant also emphasized the resistance to EBF indicating,*

*“They [first-time mothers’ family] do not accept it at all; unless the young woman tells them she must follow what the nurses have told her to do. However, her mother-in-law will grumble saying they do not have any good food to eat, how can the baby feed only on breast milk? If she goes out and leaves her baby behind, they will give it water to drink”* **(*IDI, Community 4*).**

### Socio-economic factors influencing breastfeeding practices

Culminating from the personal experience and affirmation from others, first time mothers observed EBF as economical, accessible, nutritious, as well as a means for promoting good health. Knowledge of the benefits of EBF led to an emerging trend, where some young mothers were re-oriented, and thus accepted EBF. One mother for instanced stated that *“I have seen babies whose mothers give them water and are running diarrhoea. They are always sick and their mothers are always running to the hospital” (****Mother, FGD, Community 1)****. A woman in another community corroborated this assertion indicating that “When I delivered and breastfed my baby with only breast milk, he was healthy and fine looking. Even at the A&C [antenatal clinic] the nurses said he was looking fine. So other women should also practice the exclusive breastfeeding for it is good for the children”* ***(Mother, FGD, Community 2).***

However, most women in the Kassena-Nankana Municipality engaged in productive roles (i.e. tasks which are undertaken to get paid) simultaneously with the usual reproductive roles of childbearing and housekeeping. Thus, majority of the mothers interviewed were farmers, artisans and traders who could not afford to stay home all day to breastfeed their infants at the expense of these productive roles. In such cases, the need for supplementary food was necessary, leading to a rise in the demand for alternative milk for the child’s feeding in the absence of the mother. This assertion can be supported by a mother’s statement below:

*“You know we have two types of milk, powdered milk and liquid milk. So, in my opinion, you should buy the liquid milk and keep so that when you are not around, they can feed the baby with that one” (****Mother, FGD, Community 3).***

Similarly, the performance of other reproductive roles including house chores could render mothers unavailable to breastfeed always and this supports the argument made by one mother that *“If you leave your baby behind and go to fetch water and he/she cries, the throat will dry and he/she may die.” (****Mother, FGD, Community 4)***. This presupposes that EBF was seen as a threat to the baby’s life, making the utilization of alternative milk or water a preferred choice for infant feeding in the mothers’ absence.

## Discussion

Exclusive breastfeeding is known to have diverse relevance to the mother and the baby, and its practice is a necessary condition in achieving global health targets [22]. In this article, a number of factors that compel young mothers either to undertake EBF practice or to default EBF protocol, have been highlighted. The findings are therefore relevant in advancing knowledge critical in strengthening the implementation of EBF in Ghana and other LMICs, thus contributing to curbing infant malnourishment, child mortality and enhancement of maternal health.

Among the drivers for EBF practice in Kassena Nankana was the availability of accurate information on EBF. The findings indicate that the health facility or place of delivery was the first point of call in receiving information on breastfeeding. This is therefore consistent with the findings of a study conducted in Southern Nigeria where mothers reported often obtaining EBF information when they attended antenatal clinics in health facilities [31]. On a whole, participants knew EBF as a standard practice, which makes no room for alternative feeding for babies within the specified six-month period.

In addition, perceptions formed around EBF was found to influence the practice. First-time mothers felt encouraged to feed their infants with breastmilk alone due to perceived benefits of the practice. As have been recognized in other studies [1, 5], the current study among other benefits, revealed EBF as an act which served as an immunity for the child, an effective intervention against infections, and enhanced the immediate and future health of the infant. Again, the current study has also shown EBF as an effective measure for birth spacing as well as contributing to the personal wellbeing of the mothers, confirming previous research [6–10]. First-time mothers who positively perceived EBF practice as beneficial were more likely to practice it.

In addition, we examined the underlining socio-economic factors that influence the practice of EBF. The findings show that working first-time mothers face the challenge of performing their reproductive and productive roles concurrently. In some cases, mothers working on farms or employed had to choose either to abandon their economic duties in order to breastfeed exclusively, or vice versa. The choice to abandon EBF by these first-time mothers resulted from the impracticality of taking the newly born to their places of work. This finding concurred with that of a previous study which reported that, countries with guaranteed breastfeeding breaks at work had 71% of its working mothers practicing exclusive breastfeeding while only 4% of working mothers practiced EBF in countries with unpaid guaranteed breaks [32]. Similarly, Cripe reported on the challenge faced by working mothers, indicating that some mothers hardly return to work after delivery since they believe working and breastfeeding are incompatible [33]. Notably, these previous studies were conducted among women working in the formal sector of the economy, whereas the present study was conducted among women taking active roles primarily in the informal sector. It can therefore be argued that a mother’s choice to either practice or abandon EBF is independent of the kind of job. However, the availability of time to breastfeed and the provision of enabling environment at the workplace were critical [33, 34].

In addition, the culture in which people live and are socialised is found to impact their behaviour [35]. In a collective cultural orientation, like that of Ghana, where a person is positioned in a complex network of social relationships, the intention of a mother to exclusively breastfeed is inadequate [36]. In the same view, Sulaiman and his peers, have also indicated that intentions to a lesser extent influence breastfeeding practice [34]. Other cultural barriers such as the performance of cultural rites for both mother and baby, as well as the influence of family members impacted EBF rates [1, 9, 19, 34, 37, 38]. Therefore, the statement that, “Once the child is delivered, he is no more your baby”, confirms the practice of a collective culture in the Kassena-Nankana Municipality and explains the cultural restrictions against mothers’ intention to breastfeed exclusively.

Evidently, health service-related factors such as receiving consistent feeding advice, being encouraged by maternity staff to breastfeed at birth, demand feeding while in hospital, and early breast contact (< 30 min after birth) were positively associated with exclusive breastfeeding after hospital discharge [37, 39]. The discussion with the mothers also indicated that the knowledge and perceptions of mothers and their relatives in Kassena-Nankana area was changing due to increase of information on EBF from health care providers. The increase in awareness of EBF and its implications, to some extent, led to a change in trend, where enlightened community members begin to accept EBF practices. Similarly, Aborigo in his study in Northern Ghana, pointed out that perceptions on most traditional ways of feeding infants are giving way to current recommendations [40].

Despite these interesting findings, the basic limitation in the conduct of the study is the subjective researcher positionality. In addition, the findings are specific to the Kassena Nankana locality and may not be generalizable to other settings. However, these did not influence the quality and credibility of data obtained.

## Conclusion

The choice to exclusively breastfeed result from the complex interaction of structures within which the woman is embedded. The study revealed that first-time mothers and their family members had received information on EBF. Essentially, the knowledge of the benefits of EBF to mother and child shaped the perceptions of first-mothers and motivated them to pursue the act. However, the mother’s intension to breastfeed, was found to be inadequate in the decision-making process regarding child feeding. A number of socio-cultural and economic factors were identified as contributing factors that explain breastfeeding practices in the Kassena-Nankana Municipality. Notably, the economic engagement of lactating mothers could serve as a barrier even when decision to breastfeed have been made. The availability of time to breastfeed is a critical factor. Again, the internalization of EBF information received from community health care providers in Kassena-Nankana Municipality has among others, contributed to the emerging positive trend of the acceptability of EBF practice.

## Supplementary information

**Additional file 1.**

**Additional file 2.**

## Data Availability

The datasets used and analyzed during the current study are available from the corresponding author on reasonable request.

## Abbreviations

CHPS: Community Health-based Planning Services compounds
EBF: Exclusive Breastfeeding
FGD: Focus Group Discussion
GDHS: Ghana Demographic and Health Survey Report
IDI: In-depth Interview
LMICs: low and middle-income countries
UNICEF: United Nations Children’s Fund
WHO: World Health Organization
