# Supplementary material for: “Once the child is delivered, he is no more your baby,” Exclusive Breastfeeding experiences of first-time mothers in Kassena-Nankana Municipality, Ghana - a qualitative study
Source: BMC Pregnancy Childbirth. 2020 Sep 29;20:575. doi: 10.1186/s12884-020-03272-5 (PMC7526357; doi:10.1186/s12884-020-03272-5)
Supplement: Supplementary file 1 — Additional file 1. [file 12884_2020_3272_MOESM1_ESM.doc]

# FOCUS GROUP DISCUSSION GUIDE FOR FIRST-TIME MOTHERS IN

**KASSENA-NANKANA MUNICIPALITY**

**TOPIC:** EXCLUSIVE BREASTFEEDING PRACTICES AMONG FIRST-TIME

MOTHERS IN KASSENA-NANKANA DISTRICT

**SCREENER FOR FIRST-TIME MOTHERS**

##### Background information of focus group discussion members

**Name of Community………………………….**

| 1. Age at last birthday ……. |  |  |
| --- | --- | --- |
| 1. Ethnicity    1. Kassena    2. Nankana    3. Others      1. Religion    1. Christian    2. Muslim    3. Others      1. Occupation    1. Civil servant |  | 1. Level of education    1. No formal education    2. Primary    3. JSS    4. SHS    5. Tertiary 2. Marital status    1. Single    2. Married    3. Unmarried    4. Widowed |

1. Unemployed (artisans)………..

e) Divorced

1. Public servant

f) Cohabiting d) Trader

7. Age of child in months…………

##### SECTION B: Knowledge and perception of Exclusive breastfeeding

1. Have you ever heard about exclusive breastfeeding? When did you first hear of exclusive breastfeeding (before or after pregnancy)? Probe for where they got the information (**sources**; community health worker, antenatal clinics, colleagues, family members etc.) and what information did they receive? Can you explain exactly what EBF

means?...............................................................................................................

………………………………………………………………………………………………

1. Did you practice exclusive breastfeeding? Probe. Why or why not?......................

………………………………………………………………………………………………

1. How long did you breastfeed your child before introducing foods. Why or why not?

(Probe on what kind of foods were given or not given)……………………………….

………………………………………………………………………………………………

1. How long do you think a mother should breastfeed her baby on only breast milk before introducing other foods? Why is it so??? Probe for reasons, benefits, advantages and

disadvantages………………………………………………………….

##### SECTION C: Exclusive breastfeeding practices

1. What should a baby 0-6 months be fed on? Probe for reasons for methods of feeding and how often is an exclusively breastfeed baby supposed to be breastfed in a day?

………………………………………………………………………………………………

1. How often did you breastfeed your baby in a day? Why or why not? (Probe for morning, afternoon and evening)…………………………………...........................

……………………………………………………………………………………….

1. When did you introduce food to your baby? Probe for why or why not and what did you feed your baby on? Probe for types of food (water, kooko, and other liquids). In your own opinion would you say your baby is growing well? Can you explain further why you are saying he/she is growing well (probe for age he/she started sitting, crawling, walking etc).

Why are you saying he/she is not growing well?

………………………………………………………………………………………………

1. Are there any cultural practices on breastfeeding in this community that you are aware of? What are they? Probe further for influence of cultural practices on exclusive

breastfeeding practices?...................................................................................................

………………………………………………………………………………………………

1. What do your relations think about exclusive breastfeeding? Why? Probe for partner, in-

laws, friends and other family members……………………………………………

………………………………………………………………………………………………

1. What do you think can be done to promote exclusive breastfeeding?.................... ......................................................................................................................................................

##### SECTION D: Challenges and coping strategies in relation to Exclusive breastfeeding

1. For those of you currently practicing EBF, can you please share your experiences with

me? How is it like? What are your likes or dislikes about it?.......................................

………………………………………………………………………………………………

………………………………………………………………………………………………

1. What are the challenges you faced with exclusive breastfeeding? Probe for challenges with self, baby, husband, friends and other family members………………………….

………………………………………………………………………………………………

………………………………………………………………………………………………

1. What did you do when you were faced with these challenges? Probe for how the managed (Probe for who supported them during the challenge and when was support

given). …………………………………………………………………………………
