# Supplementary material for: “Once the child is delivered, he is no more your baby,” Exclusive Breastfeeding experiences of first-time mothers in Kassena-Nankana Municipality, Ghana - a qualitative study
Source: BMC Pregnancy Childbirth. 2020 Sep 29;20:575. doi: 10.1186/s12884-020-03272-5 (PMC7526357; doi:10.1186/s12884-020-03272-5)
Supplement: Supplementary file 2 — Additional file 2. [file 12884_2020_3272_MOESM2_ESM.docx]

**IN-DEPTH INTERVIEW GUIDE FOR TRADITIONAL BIRTH ATTENDANTS (TBAs)**

**TOPIC:** EXCLUSIVE BREASTFEEDING PRACTICES AMONG FIRST-TIME

MOTHERS IN KASSENA-NANKAN MUNICIPALITY

**KEY INFORMANT ………….. Name of Community………………**

**Background information**

1. Sex:…………..….
2. Age:………………….
3. Marital status:…………………..
4. Educational level:………………….
5. Occupation:………………………….
6. What is your source of training? Have you received any other extra training with

regards to being a TBA? Where?................................................................................

1. How long have you been a TBA or practicing

.....................................................................................................................

1. What are the activities you do as a TBA?....................................................................

**Knowledge and Perception of Exclusive breastfeeding**

1. Do you counsel mothers on breastfeeding? Probe for why or why not? ………….

………………………………………………………………………………………

………………………………………………………………………………………

1. Have you ever heard about exclusive breastfeeding? When did you first hear of exclusive breastfeeding (before or after you started practicing)? Probe for where they got the information (**sources**; community health worker, antenatal clinics, colleagues, family members etc.) and what information did they receive? Can you

explain exactly what EBF means? .

………………………………………………………………………………………

1. What do you know about exclusive breastfeeding? Probe for benefits, consequences..

………………………………………………………………………………………

1. How long do you think a mother should breastfeed her baby on only breast milk

before introducing other foods? Why?........................................................................

………………………………………………………………………………………

1. How often is a baby supposed to be breastfed in a day? Probe for feeding at 0-6

months………………………………………………………………………………

**Exclusive breastfeeding practices**

1. What is your understanding of exclusive breastfeeding? Who do you recommend it

for; first-time mothers, multi-gravida etc? Probe for why?

(Reasons)………………….

1. In your opinion do you think a first-time mother should exclusively breastfeed? Why or why not? Probe for types of food (water, kooko, and other liquids)……………….

………………………………………………………………………………………

1. When do first time mothers in this community usually introduce food to their baby? Why or why not? Probe for types of food (water, kooko, and other

liquids)………………………………………………………………………………

……………………………………………………………………………………… ………………………………………………………………………………………

1. Are there any cultural practices about exclusive breastfeeding and first-time mothers? What are they? Probe further for influence of cultural practices on

exclusive breastfeeding practices?

..............................................................................................

1. What do relations of first-time mothers think about exclusive breastfeeding? Probe

for partners, in-laws and other family members……………………………………………

......................................................................................................................................

1. What do you think can be done to promote exclusive breastfeeding among first time

mothers?......................................................................................................................

**Challenges and coping strategies in relation to Exclusive breastfeeding**

1. What are the challenges faced by first-time mothers with regards to exclusive breastfeeding in this community? Probe for baby, mother, partners and other family members. What are the complaints that you get from first-time mothers regarding exclusive breastfeeding? What advice do you give them? Do you give any specific support?

.......................................................................................................................

………………………………………………………………………………………

1. What do they do when they are faced with these challenges? And what do you

recommend that they do. Probe for why?....................................................................

**IN-DEPTH INTERVIEW GUIDE FOR HEALTHWORKERS**

**TOPIC:** EXCLUSIVE BREASTFEEDING PRACTICES AMONG FIRST-TIME

MOTHERS IN KASSENA-NANKANA DISTRICT

**KEY INFORMANT ………….. Name of community……………….**

**Background information**

1. Sex:
2. Occupation(professional background):……………………
3. Marital status:…………………..
4. Educational level:………………….
5. How long have you been practicing?...................................................
6. What activity does your occupation entails?...........................................

**Knowledge and Perception of Exclusive breastfeeding**

1. Do you counsel mothers on breastfeeding? Probe for why or why not?.....................

......................................................................................................................................

......................................................................................................................................

1. Have you ever heard about exclusive breastfeeding? When did you first hear of exclusive breastfeeding (before or after you started practicing)? Probe for where they got the information (**sources**; community health worker, antenatal clinics, colleagues, family members etc.) and what information did they receive? can you explain what EBF means

………………………………………………………………………………………

………………………………………………………………………………………

1. What do you know about exclusive breastfeeding? Probe for benefits, consequences

………………………………………………………………………………………

………………………………………………………………………………………

1. How long do you think a mother should breastfeed her baby on only breast milk

before introducing other foods? Why?........................................................................ ………………………………………………………………………………………

………………………………………………………………………………………

1. How often is a baby supposed to be breastfed in a day? Probe for feeding at 0-6

months………………………………………………………………………………

**Exclusive breastfeeding practices**

1. What is your understanding of exclusive breastfeeding? Who do you recommend it for; first-time mothers, multi-gravida women etc? Probe for why?

(Reasons)

………………………………………………………………………………………

1. In your opinion do you think a first-time mother should exclusively breastfeed?

Why or why not?

................................................................................................................

1. When do first time mothers in this community usually introduce food to their baby? Why or why not? Probe for types of food (water, kooko, and other liquids)

………………………………………………………………………………………

………………………………………………………………………………………

1. Are there any cultural practices about exclusive breastfeeding and first-time mothers? What are they? Probe further for influence of cultural practices on

exclusive breastfeeding practices?

………………………………………………………………

1. What do relations of first-time mothers think about exclusive breastfeeding? Probe

for partners, in-laws and other family members……………………………………………

………………………………………………………………………………………

1. What do you think can be done to promote exclusive breastfeeding among first timemothers?................................................................................................................

**Challenges and coping strategies in relation to Exclusive breastfeeding**

1. What are the challenges faced by first-time mothers with regards to exclusive breastfeeding in this community? Probe for baby, mother, partners and other family members. What are the complaints that you get from first-time mothers regarding exclusive breastfeeding? What advice do you give them? Do you give any specific support?

..................................................................................................................

1. What do they do when they are faced with these challenges? And what do you

recommend that they do. Probe for why?................................................................ ………………………………………………………………………………………
